# Treatment and Preservation of the Deciduous Teeth

**Published:** 1870-07

**Authors:** E. M. Morrison


### TREATMENT AND PRESERVATION OF THE DECIDUOUS TEETH.

BY E. M. MORRISON, M. D.

Read before the Indiana State Dental Association, June 29, 1870.

This is a subject that many of us do not have much to do with, but we should all be well informed as to the best methods of treatment and preservation of these organs, which have so much to do with the health in the transition periods of life, from birth to maturity. The deciduous teeth are the principal masticators during the early portion of these periods, and the development of the physical system depends, to a considerable extent, upon their health, and the proper performance of their functions. The mind also has a great dependence here; for the difficulties and diseases incident to dentition are often productive of permanent injury to the cerebral functions.

Dr. Gross says: "A very lively sympathy exists between the teeth and some other parts of the body; more intimate and extensive than would at first sight seem possible. Children, from the pressure of the teeth upon the gums, are extremely liable, especially during our hot summer months, to vomiting, diarrhœa, fever, and convulsions. Arachnitis occasionally supervenes upon difficult dentition; and certain affections of the skin, as eczema and porrigo, are frequently directly traceable to its effects, and rendered obstinate, if not temporarily incurable, by its presence."

Then, this subject should demand our earnest consideration; but I am sorry that my opportunities for observation have not been very extensive, or of a well marked character; so you will have to look to those among us whose opportunities have furnished them better means of observation, to furnish you with the material for discussion.

The eruption of the deciduous teeth is one of the first steps of independent life; and in their development, duration, and decay, the periods of infancy, childhood, and youth may be marked, and when the last of them is shed the youth may be said to merge into maturity.

For the time of eruption of the deciduous teeth, you are referred to Carpenter, or other standard authors. This period varies from the eighteenth to the thirty-sixth month; and Dr. Carpenter says it is one of considerable risk to the infant's life. The great mortality of this period also shows the fact; and Meigs and Pepper speak of dentition in infants as being an active agent in the production of entero colitis, cholera infantum, eclampsia, etc.; and say: "Attention to the state of the gums should never be neglected in teething children. * * * If teeth are felt distinctly through the gums, and the gums be swollen, tense, hot, and highly vascular, cut them freely, once. If, on the contrary, the gums are firm, not hot, not redder than usual, and the edges of the teeth can not be felt, it is foolish meddlesomeness to cut them." Dr. Gross, however, offers a mitigation for this meddlesomeness, but no justification of it. He says: "It has been objected to this operation that, when it is not followed by the immediate extrusion of the teeth, the cicatrice that will form over it by the healing of the gum, will afterwards render its eruption more difficult; but such a conclusion is alogether erroneous, as all new tissues are much more easily destroyed than old or preexisting." He also insists that: "The proper remedy for difficult dentition is free division of the gums, and the inclosing membrane of the advancing tooth." Dr. Marshall Hall recommends the free use of the gum lancet, as a means of prevention and cure in the convulsive affections of infants teething; but Dr. Carpenter believes that we should pay more attention to the general system than is generally bestowed in such cases. Too much reliance has been placed upon the gum lancet, and local treatment, in many cases, and not enough on the condition of the general system; yet we are forced to admit that the gum lancet is a most important remedial agent; but we can not rely upon it as a safe prophylactic, for it has been shown by experience, and by authors, that in a state of health we need have but little fear about dentition. It is a physiological process, and nature has made provision for it without the aid of surgery. When the necessity arises, however, I do not hesitate to use the gum lancet freely; but I would not think of excluding other remedial agents to correct whatever complication, or cause of disturbance may exist at the time. While I was in the general practice this class of cases was frequently met with; and my success in their treatment was fair; but now that I have advanced to the specialty of a dentist, they do not often come under my immediate care, or control.

After the eruption of the teeth the treatment and means of preservation, will depend on circumstances, or on the state of health at the time. We will take rickets as an example. It is a well known fact that the development of the teeth is retarded in this disease, and that those already erupted soon become carious, and are of short duration.

Dr. Smith says: "If the disease makes its appearance before any of the teeth are cut, their evolution may be almost indefinitely postponed. * * * Teeth which have already appeared, speedily become black, decay, and drop early from their sockets." According to Dr. Vogle, this is due to insufficient development of the dental enamel, and it is common to see rickety children, of eighteen months, or two years, with but few teeth, and those few black and carious. Dr. Hillier says: "The teeth are often deficient in enamel, so that they crumble, and become dark in color."

In cases of this kind it is plain that local treatment can not be remedial. We must go further than this, and reach the constitutional requirements, or we can not expect success in our treatment of the teeth. The dentist who is only a "rubber boiler," must stand aside for better skill, and acknowledge the supremacy of a higher order in his profession. He is a failure, a mere parasite, to leech out our life-blood, and cast reproach on the profession. If, on the other hand, he comprehends the case fully, he will see the indications to be fulfilled, and succeed in most cases. This disease, says Dr. Smith, "Softens and consumes the osseous tissues, so that the bones lose in density, in weight, and in firmness;" and it is claimed that this softening is due to the removal of the lime salts, which enter the blood in a soluble form, and are excreted by the kidneys.

The treatment should consist of good diet, suited to the degree of debility, fresh air, good flannel under-clothing, cleanliness, and suitable tonics, as iron, quinine, cod liver oil, etc. This course will generally effect a cure of the rickets, but the tooth deformities, and losses by caries, can not thus be rebuilt, yet the foundation for a better permanent set may be improved. We should, in this case, especially, as in all others, endeavor to restore and maintain the best possible conditions as to the general health. When this is done, and we have secured the enforcement of proper rules for cleanliness and care of the teeth we have but little else to see after.

The dentist, or physician, however, is not generally called upon till the teeth are carious, or there is aching, periostitis, or dental fistula, and the little sufferer has fallen sick from the accumulation of maladies caused by direct inflammation, or through sympathy. Dr. Gross gives a number of instances in which the marked influence of sympathy is manifested, but we have seen these so often in adults that we are not likely to be misled by them; and I need not recite them here; yet I will say that when an infant, or child, has pain in the face, neck, or ear, or has enlargement of the lymphatic glands of the neck, or any kindred affections, we should look well to the condition of the mouth and teeth. If they are diseased, which they are most likely to be, we should restore them to health as speedily as possible. If caries exists in any case, and has not progressed too far, we should fill the teeth in the best manner we can; but if this can not be done, and the teeth ache severely, I would not hesitate to extract them; or if we have necrosis, or if a fistula exists, I would, as a rule, extract; for in relation to the child's health, and the development of the permanent teeth, I would fear the consequences of these diseases more than I would that of extraction. In this course I have good medical authority Dr. Gross, in speaking of dental fistula, says: "The most effectual remedy for this affection is the prompt removal of the offending tooth;" and recommends extraction for carious teeth, that can not be filled, and for necrosis. Dr. Carpenter says: "The 'milk' teeth originate, or are developed, from mucous membrane. The 'permanent' teeth, also originating from mucus membrane, are of independent origin, and have no connection with the 'milk' teeth." So, on these grounds, I claim that we are justified in this course of treatment, as being the best and surest of success.

Some Dentists fear to extract the deciduous teeth, on various considerations, as: injury to the permanent teeth,—crowding; and deformity of the jaws; to some extent these objections are good, but when a multiplicity of evils are before us, without the possibility of averting all of them, we should choose the least. Upon this theory I have aimed to base my practice, believing that the causes of irritation and inflammation, so rife and dangerous to the infantile periods of life, should be removed as speedily as possible, and with the greatest certainty of success.

It is good practice, in all cases where it is at all practicable, to fill the teeth, but if we depend on our gold or tin, and do not restore the general health we will most likely be mortified by failure. In rickets it is probable that the chances for success are the poorest, for there is no other disease in which the development of the teeth is arrested, or that the structure is so much changed or weakened, as in this, but the treatment to be adopted must be suited to the indications, to be fulfilled in each individual case. We have all seen many failures in fillings for adults, for want of the proper restoration to health of parts in contact, or in sympathy with the teeth filled.

In children the deciduous teeth are much more susceptible to external infirmities, for want of durable structures; and on account of fluctuations in habits and health there is a more lively sympathy manifested at this time of life, than in adult age, and in proportion to the extent and intensity of these influences, and the feebleness of the reasoning faculties of children, we have the difficulties in the treatment and preservation of the deciduous teeth augmented. We may treat the deciduous teeth, however, on the same general principles that we do the permanent, but we must ever bear in mind that they are deciduous, and that when diseased they are most potent causes in the production of convulsions, and the dangerous inflammatory and digestive diseases of children; and that it is often better to sacrifice a tooth thus diseased, prematurely, than to take the risk that its presence in the mouth will necessarily involve.

